# Thrombose bilatérale des veines subclavières: une manifestation exceptionnelle de la sclérodermie systémique

**DOI:** 10.11604/pamj.2015.20.393.6801

**Published:** 2015-04-21

**Authors:** Olfa Berriche, Wafa Chebbi

**Affiliations:** 1 Service de Médecine Interne, CHU Taher Sfar Mahdia, 5100 Mahdia, Tunisie

**Keywords:** Sclérodermie systémique, thromboses veineuses profondes, anticoagulation, systemic sclerosis, DVT, anticoagulation

## Image en medicine

La sclérodermie systémique est une affection généralisée du tissu conjonctif, des artérioles et des micro-vaisseaux, caractérisée par la survenue de phénomènes de fibrose et d'oblitération vasculaire. Les thromboses veineuses profondes représentent une manifestation exceptionnelle de la sclérodermie systémique. Le dysfonctionnement endothélial, les anomalies de régulation des fibroblastes à l'origine d'un excès de synthèse du collagène et le dérèglement immunitaire, observés au cours de la sclérodermie systémique, serait plutôt en faveur de caractère non fortuit de cette association et explique en partie le caractère thrombogéne de la sclérodermie systémique. Nous rapportons l'observation d'une patiente âgée de 56 ans, hospitalisée pour un œdème du visage d'apparition brutale avec un comblement des creux sus claviculaires. Elle était suivie depuis 4 ans pour une sclérodermie systémique dont le diagnostic était retenu devant la présence d'une sclérodactylie, une sclérose proximale, un syndrome de Raynaud avec des mégacapillaires à la capillaroscopie, et une atteinte digestive à type de dysphagie mixte avec à la manométrie une hypotonie du sphincter inferieur de l’œsophage. La patiente était traitée par un inhibiteur calcique, de la colchicine et inhibiteur de la pompe à proton. L’échographie doppler des membres supérieurs demandée en urgence montrait une thrombose bilatérale des veines subclavières. Le bilan étiologique de la thrombose veineuse était négatif (hémogramme normal, absence de lésion tumorale, taux sanguins normaux de protéine C, d'anti-thrombine, de protéine S, de résistance à la protéine C activée et d'homocysteine, anticorps antiphospholipides négatifs). La patiente était mise sous anticoagulant avec une évolution favorable.

**Figure 1:**
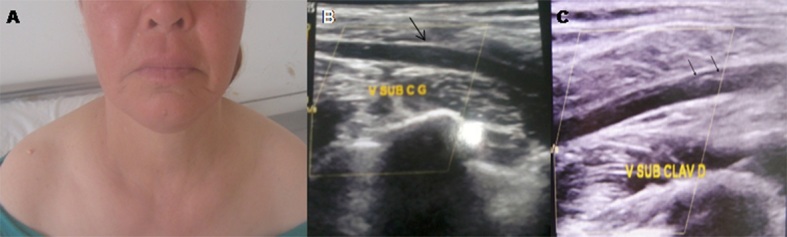
(A) comblement des creux sus claviculaires. Echographie Doppler des membres supérieurs: absence de flux au niveau des veines subclavières (B) gauche, et (C) droite; qui sont dilatées avec un contenu échogène (fleches)

